# Role of MicroRNA-1 in Human Cancer and Its Therapeutic Potentials

**DOI:** 10.1155/2014/428371

**Published:** 2014-05-18

**Authors:** Chao Han, Zujiang Yu, Zhenfeng Duan, Quancheng Kan

**Affiliations:** ^1^Department of Clinical Pharmacology, The First Affiliated Hospital of Zhengzhou University, Zhengzhou 450052, China; ^2^Sarcoma Biology Laboratory, Center for Sarcoma and Connective Tissue Oncology, Massachusetts General Hospital, Boston, MA 02114, USA

## Abstract

While the mechanisms of human cancer development are not fully understood, evidence of microRNA (miRNA, miR) dysregulation has been reported in many human diseases, including cancer. miRs are small noncoding RNA molecules that regulate posttranscriptional gene expression by binding to complementary sequences in the specific region of gene mRNAs, resulting in downregulation of gene expression. Not only are certain miRs consistently dysregulated across many cancers, but they also play critical roles in many aspects of cell growth, proliferation, metastasis, apoptosis, and drug resistance. Recent studies from our group and others revealed that miR-1 is frequently downregulated in various types of cancer. Through targeting multiple oncogenes and oncogenic pathways, miR-1 has been demonstrated to be a tumor suppressor gene that represses cancer cell proliferation and metastasis and promotes apoptosis by ectopic expression. In this review, we highlight recent findings on the aberrant expression and functional significance of miR-1 in human cancers and emphasize its significant values for therapeutic potentials.

## 1. Introduction

**Figure 1:**
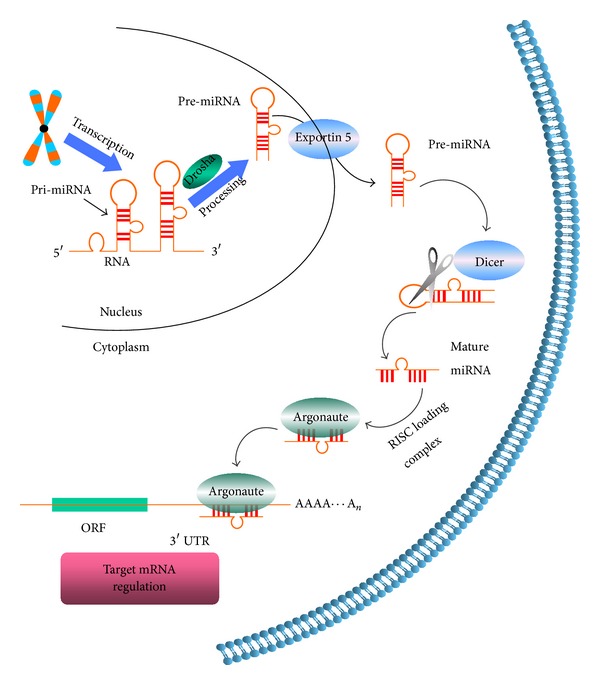
Biological principles of miRNA (miR). miR genes are transcribed by RNA polymerase II (Pol II) to generate the primary transcripts (pri-miRNAs). The following step is mediated by the Drosha complex that generates ~65 nucleotide (nt) precursor-miRs (pre-miRNAs). Pre-miR has a short stem plus a ~2-nt 3′ overhang, which is recognized by the nuclear export factor exportin 5. On export from the nucleus, the cytoplasmic RNase III Dicer catalyzes the second processing (dicing) step to produce miR duplexes. One strand of the duplex pre-miR remains as the mature miR, whereas the other strand is degraded. Dicer and Argonaute mediate the processing of pre-miR and the miR mature strand is then assembled into the RNA-induced silencing complex (RISC) and targets the complementary mRNA 3′-UTR sequences via translational repression or mRNA cleavage.

MicroRNAs (miRNAs, miRs) are a subset of small noncoding RNA molecules that are approximately 22 base pairs in length. miRs play important roles in many aspects of cell biology, including cell fate determination, metabolism, stress responses, apoptosis, and carcinogenesis [1–4]. miRs were first discovered in 1993 during a study of* Caenorhabditis elegans* development, associated with the gene lin-14 [5]. The evolution of most miRs is very conservative and often found in clusters [6]. Primary miRs are produced by RNA polymerase II (Pol II) in a stem-loop structure and then cropped to form precursor miR (pre-miR) hairpins in the nucleus and cytoplasm by a multiprotein complex that includes Drosha and Dicer. Finally, the pre-miRs are cleaved by Dicer to produce mature miRs [7–9]. Mature miRs mainly bind to the 3′-untranslated regions (UTRs) of target genes and inhibit gene expression by degradation or repress translation of the target mRNA (Figure 1) [10, 11]. So far, more than 2,500 human miRs have been registered at miRBase in release 20.0 (http://microrna.sanger.ac.uk/). Bioinformatics predictions indicate that miRs regulate more than 60% of protein-coding genes [10].

Recently, the function of miRs in human cancers has been of interest to many [12–14]. Numerous studies have demonstrated dysregulation of miRs in tumors by microarray assay [15, 16]. The aberrant expression of miRs is mainly divided into two classes: upregulated miRs and downregulated miRs. Low expression of miRs in cancer has been reported to repress or downregulate the expression of oncogenes, resulting in the inhibition of cancer cell proliferation [17, 18]. Other highly expressed miRs in cancer have been shown to inhibit tumor suppressor genes and promote tumor proliferation and metastasis [19, 20]. Although specific miRs are overexpressed in cancer cells, most are downregulated in tumors, thus suggesting the possibility of more tumor suppressing miRs than oncogenic miRs [21, 22]. Among them, miR-1 is the most consistently decreased miR in various human cancers, suggesting the great potential miR-1 replacement therapy holds for cancer treatment.

## 2. Basic Biology of miR-1 and Validated Functions of miR-1 in Normal Tissues

**Figure 2:**
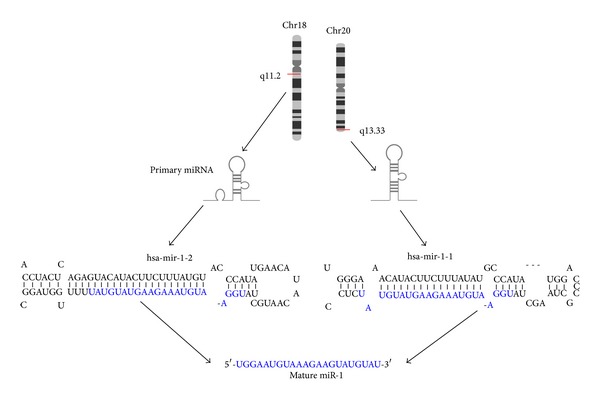
Alignment of two different precursors (hsa-miR-1-1 and hsa-miR-1-2) of miR-1 in human. hsa-miR-1-1 and hsa-miR-1-2 are located in two distinct chromosomal regions in human genome, −20q13.33 and 18q11.2. Both these precursors are processed into an identical mature form of miR-1. The respective pre-miR-1 sequences (hsa-miR-1-1 and hsa-miR-1-2) were listed. Each sequence is shown with blue characters indicating mature miR-1 nucleotides.

There are two different precursors of miR-1 in human, miRNA-1-1 and miRNA-1-2, both of which are processed into an identical mature form of miR-1. miRNA-1-1 and miRNA-1-2 are located in two distinct chromosomal regions in human genome, −20q13.33 and 18q11.2, respectively (Figure 2). Inside the cell, miR-1 is transcribed as ~70 nucleotide precursors and subsequently processed by the Dicer enzyme to give ~22 nucleotide mature products. The mature sequence comes from the 3′ arm of the miR-1 precursor. Literature review suggests miR-1 to be expressed specifically in the normal cardiac and skeletal muscle tissues. Upon induction of myogenic differentiation, miR-1 was highly expressed. Retrovirus-mediated overexpression of miR-1 markedly enhanced expression of muscle creatine kinase, sarcomeric myosin, and alpha-actinin, while the effects on myogenin and MyoD expression were modest. Formation of myotubes was significantly augmented in miR-1-overexpressing cells, indicating that miR-1 expression enhanced both myogenic differentiation and maturation into myotubes [23]. A more recent study showed that miR-1 induction can directly downregulate Notch3 and allow differentiation of myoblast cells [24], suggesting that myogenic differentiation-induced miR-1 is critical for differentiation at least partly by turning off Notch3. Notch3 serves as a regulator for preventing premature myogenic differentiation [24].

## 3. Expression of miR-1 in Noncancer Human Diseases

miR-1 has crucial functions in the development and physiology of muscle tissues and related diseases [25, 26]. Studies have shown abnormal miR-1 expression in heart diseases including myocardial infarction, arrhythmias, and heart failure [27–29]. Reduction in expression of miR-1 has been found in serum of patients with chronic obstructive pulmonary disease (COPD), and miR-1 is downregulated in myocardial infracted tissue [30]. Expression of miR-1 was associated with smoking history and lung function. Furthermore, the protein levels of histone deacetylase 4 (HDAC4), a miR-1 target gene, were increased in the patients with COPD. miR-1 expression was negatively correlated with phosphorylation of the Akt kinase [31]. These data suggest that expression of miR-1 may contribute to COPD-associated skeletal muscle dysfunction.

## 4. Aberrant Expression of miR-1 in Human Cancers

Using high-throughput miR microarray technology and quantitative RT-PCR (qRT-PCR) for validation, many studies have found decreased expression of miR-1 in various types of human cancers (Table 1). Associations between miR-1 expression levels and tumor type, grade, response to treatment, and prognosis have also been reported in recent studies. We will review the expression of miR-1 and its potential functions in lung cancer, gastrointestinal cancer, genitourinary cancer, head and neck cancer, and sarcoma.

### 4.1. miR-1 in Lung Cancer

Lung cancer is the leading cause of cancer-related deaths in men and women in the world [32]. miR-1 has been shown to be significantly downregulated in vinyl carbamate- (VC-) induced mouse lung tumor models [33]. miR-1 expression is not only significantly reduced in primary lung cancer tissues but also in many lung cancer cell lines [34–36]. miR-1 has been shown to be antitumorigenic in lung cancer cells [34, 36, 37]. Expression of miR-1 in non-miR expressing lung cancer cells reversed their tumorigenic properties of growth, replication potential, motility/migration, clonogenic survival, and tumor formation in nude mice [34]. Exogenous miR-1 significantly reduced expression of oncogenic targets, MET and Pim-1. MET and Pim-1 are frequently upregulated in lung cancer and promote the growth and survival of cancer cells. Similarly, the levels of two additional targets, FoxP1, a transcription factor with ontogenetic property, and HDAC4, that represses differentiation-promoting genes, were also reduced in miR-1-expressing cells. Conversely, depletion of miR-1 increased cell growth with associated elevation of these target genes [34]. In a recent study of serum miR signature to predict survival of non-small-cell lung cancer (NSCLC), miR-1 level in serum was found to be significantly lower in shorter-survival group as compared with longer-survival group, and levels of miR-1 were significantly associated with overall survival [38]. Meanwhile, one study about miR-1 and NSCLC found that almost 70% of the NSCLC tissue samples showed low miR-1 expression and high PIK3CA expression [35]. Moreover, there is a significant relationship between the expression levels of miR-1 and PIK3CA in NSCLC and clinical characteristics and prognosis [35]. The results show that not only the patients with low miR-1 expression but also those with high PIK3CA expression had significantly higher incidences of lymph node metastases and recurrences in one year after surgery than other patients. So, the expression levels of miR-1 and PIK3CA in NSCLC tissues may be useful for predicting lymph node metastasis and postoperative recurrence in patients with NSCLC [35]. In another study, lung cancer A549 cells overexpressing miR-1 exhibited a significant morphological change from a mesenchymal to an epithelial phenotype characterized by cell polarization and intercellular junctions [37]. The cells showed increased expression of E-cadherin, which colocalized with cortical actin filaments and vinculin to form typical adherens junctions at the apical regions of intercellular borders. Moreover, the cells migratory and invasive activities were decreased, and their sensitivity to doxorubicin was increased. Interestingly, the structural and tumorigenic properties induced by miR-1 were associated with the reduced expression of Slug, which was a transcriptional repressor of E-cadherin or an inducer of epithelial-to-mesenchymal transition [37]. The E-cadherin-actin filaments and vinculin complex play a crucial role in epithelial cell-cell adhesion and in the maintenance of tissue architecture [37]. The effect of overexpressing miR-1 on the E-cadherin-actin filaments and vinculin complex may reflect the underlying mechanisms of miR-1 on cell migration and invitation.

### 4.2. miR-1 in Gastrointestinal Cancer

Gastrointestinal carcinomas are common malignant diseases worldwide. Among them, hepatocellular carcinoma (HCC) is the fifth most prevalent cancer and is the third leading cause of cancer-related death in China; colorectal cancer is the third most common cancer in the world, accounting for about 9% of all new cases of cancer. Gastrointestinal cancer carcinogenesis has been widely studied at the molecular level in recent years; however, the mechanism remains unclear. Recently, miR dysregulation has been described in different gastrointestinal cancers. miR-1 expression is significantly reduced in primary human hepatocellular carcinomas (HCCs) compared with matching normal liver tissues [39]. Ectopic expression of miR-1 in HCC cells inhibited cell growth and reduced replication potential and clonogenic survival [39–41]. FoxP1 and MET genes harbor three and two miR-1 binding sites in their 3′-untranslated regions, respectively. Expression of FoxP1 and MET was markedly reduced by ectopic miR-1 transfection. Upregulation of several miR-1 targets including FoxP1, MET, and HDAC4 in primary human HCCs and downregulation of their expression in 5-AzaC-treated HCC cells suggest their role in hepatocarcinogenesis. The inhibition of cell cycle progression and induction of apoptosis after reexpression of miR-1 are some of the mechanisms by which DNA hypomethylating agents suppress hepatocarcinoma cell growth [39]. In a human colon cancer study, miR-1 was downregulated in 84.6% of the tumors; this decrease significantly correlated with MET overexpression, particularly in metastatic tumors. Overexpression of metastasis-associated in colon cancer 1 (MACC1) and downregulation of miR-1 are associated with MET overexpression. Consistent with a suppressive role of miR-1, when ubiquitously expressed* in vitro* in colon cancer cells, reduced MET levels and impaired MET-induced invasive growth were seen [42].

In a separate study, miR expression in 80 colon tumors and 28 normal mucosa tissues was investigated [43]. This study identified 39 miRs demonstrating large fold change in expression levels including increased and decreased expression. miR-1 is downregulated in colon tumors as compared to the normal tissues. Based on the presence of microsatellite instability (MSI-H) and/or the absence of protein expression for human mutL homolog 1 (hMLH1) or the absence of microsatellite instability (MSS/MSI-L), the tumors were divided into two groups: defective DNA mismatch repair (dMMR) tumors (poor differentiation tumor) and proficient DNA mismatch repair (pMMR) tumors. dMMR means the presence of microsatellite instability (MSI-H) and/or the absence of protein expression for hMLH1 whereas pMMR means the absence of microsatellite instability (MSS/MSI-L) and the presence of normal protein expression for hMLH1. [43]. This study demonstrated that miR-1 was further decreased in dMMR tumors relative to pMMR tumors, indicating that expression of miR-1 is associated with tumor cell differentiation. miR-1 also has a tumor suppressor function in colorectal cancer by directly downregulating the MET oncogene both at RNA and protein level, and reexpression of miR-1 leads to MET-driven reduction of cell proliferation and motility, identifying the miR-1 downmodulation as one of the events that could enhance colorectal cancer progression [44]. Additionally, the reason that miR-1 is silenced in colorectal cancer is likely due to DNA hypermethylation, as miR-1 is methylated frequently in early and advanced colorectal cancer [45, 46]. In order to identify miR signatures for gastric cancer and for predicting clinical resistance to cisplatin/fluorouracil (CF) chemotherapy, a comprehensive miR microarray analysis was performed recently by using endoscopic biopsy samples. Levels of miR-1 expression have been shown to be associated with chemosensitivity [47]. These studies have identified an oncosuppressive role of miR-1 in different gastrointestinal cancers. Downregulation of miR-1 thus contributes to oncogene overexpression and to the metastatic behavior of gastrointestinal cancer cells.

### 4.3. miR-1 in Genitourinary Cancer

In genitourinary carcinomas, miR-1 is mainly dysregulated in prostate, bladder, and renal cancers. Prostate cancer is the most common cancer in developed countries, and it is the second leading cause of cancer deaths in men [48]. miR-1 is downregulated in prostate tumor tissue as compared to nontumor tissue by microarray analysis. Western blot analysis has confirmed that miR-1 represses its target gene exprotin 6 and protein tyrosine kinase 9 (also termed A6/twinfilin) on the protein level in two prostate cancer cell lines [49]. Little is known about the function of these two genes, but recent data suggest that both regulate cellular actin dynamic [49]. Similarly, another study demonstrated significantly lower expression of miR-1 in prostate cancer tissues [50]. Meanwhile, this study found 6 genes (TAGLN2, WDR78, C4orf34, PNP, LASS2, and STXBP4) had putative target sites for miR-1 in their 3′UTR by Target Scan Program [50]. PNP (purine nucleoside phosphorylase) was highly expressed in prostate tumors as compared with nonprostate tumor tissues by RT-PCR and potentially functions as an oncogene. Overexpression of miR-1 not only inhibits prostate cancer cell proliferation, migration, wounding-healing, and invasion activity but also inhibits the expression of PNP in these cell lines [50]. Another more recent study showed miR-1 is among the most consistently downregulated miRs in primary human prostate tumors [51]. miR-1 expression is further reduced in distant metastasis and is a candidate predictor of disease recurrence. Further* in vitro* experiments showed miR-1 is epigenetically silenced in human prostate cancer, although the mechanisms underlying miR-1 deregulation in cancer are not well understood. Overexpression of miR-1 in these cells led to growth inhibition and downregulation of genes in pathways regulating cell cycle progression, mitosis, DNA replication/repair, and actin dynamics [51]. A gene set enrichment analysis revealed that the miR-1-mediated tumor suppressor effects are globally similar to those of histone deacetylase inhibitors. Additional data showed evidence that miR-1 alters the cellular organization of F-actin and inhibits tumor cell invasion and filopodia formation [51]. Another interesting study used an integrated functional miR-target interaction with mRNA and miR expression to infer mRNA-mediated miR-miR interactions and revealed that miR-1 is a key player in regulating prostate cancer progression. miR-1 were identified as diagnostic and prognostic biomarkers in the 11 miRs that act as tumor suppressor miRs [52]. Epithelial-mesenchymal transition (EMT) is a developmental program of signaling pathways that determine commitment to epithelial and mesenchymal phenotypes. EMT processes have been implicated in benign prostatic hyperplasia and prostate cancer progression. The Slug gene has been shown to be a major regulator of mesenchymal differentiation. Slug is also a direct repressor of miR-1 transcription. Slug and miR-1 act in a self-reinforcing regulatory loop, leading to amplification of EMT. Depletion of Slug inhibited EMT during tumorigenesis, whereas ubiquitous expression of miR-1 inhibited both EMT and tumorigenesis in human and mouse model systems [53]. These extensive studies indicate that miR-1 acts as a tumor suppressor in prostate cancer by influencing multiple cancer-related processes and by inhibiting cell proliferation and motility.

Bladder cancer is the second most common cancer of the genitourinary tract [54]. miR-1 is downregulated in bladder cancer tissues [55]. miR-1 also functions as a tumor suppressor in bladder cancer. Transfected miR-1 into bladder cancer cell lines inhibits tumor cell viability, invasion, migration activity, and proliferation [56–58]. These effects were achieved through regulating the various miR-1 targeted genes. Moreover, expression of miR-1 has been reported to promote apoptosis. Transfection of miR-1 in bladder cancer cell lines significantly increased caspase-3/7 activities. Similar results were observed with the knockdown of miR-1 targeted gene SRSF9 [58]. In another recent study by the same group, novel molecular targets regulated by miR-1 in bladder cancer were identified [59]. Genome-wide molecular target search and luciferase reporter assays showed that prothymosin-*α* (PTMA) and PNP are directly regulated by miR-1. Silencing of these two genes significantly inhibited cell proliferation and invasion and increased apoptosis in bladder cancer cells. Immunohistochemistry showed that PTMA expression levels were significantly higher in BC compared to normal bladder epitheliums. PTMA and PNP were identified as new target genes regulated by miR-1 in bladder cancer. These genes may function as oncogenes contributing to cell proliferation and invasion in bladder cancer [59]. PTMA is one of the miR-1 target genes involved in miR-1 inducing apoptosis. Inhibition of PTMA can accelerate apoptotic progression in cells treated with anticancer drugs [59]. Therefore targeting PTMA may have potential applications as an adjuvant in cancer chemotherapy.

### 4.4. miR-1 in Head and Neck Cancer

Head and neck cancer are tumors localized in oral cavity, oropharynx, hypopharynx, and larynx. In head and neck cancers, squamous cell carcinomas account for 90%, collectively referred to head and neck squamous cell carcinoma (HNSCC). HNSCC has been ranked as one of the top ten cancers worldwide for the past 20 years and the survival rate has remained unchanged; half of the cases die within 5 years of diagnosis [32, 60]. Researchers analyzed miR expression profiles of HNSCC and adjacent normal tissues by miR microarray, which showed six miRs (miR-21, miR-1, miR-133a, miR-205, miR-206, and let-7d) exhibiting low expression levels as compared to normal specimen [61]. It is hypothesized that these miRs might distinguish between different HNSCC tumor characteristics. miR-1 also inhibits head and neck cancer cell growth by targeting different miR-1 targeted genes [62–64]. For example, studies have investigated the functional significance of miR-1 in HNSCC cells and identified miR-1-regulated novel cancer pathways. Gain-of-function studies using miR-1 revealed significant decreases in HNSCC cell proliferation, invasion, and migration. In addition, the promotion of cell apoptosis and cell cycle arrest was demonstrated following miR-1 transfection of cancer cells. A search for the targets of miR-1 revealed that transgelin 2 (TAGLN2) was directly regulated by miR-1. Silencing of TAGLN2 significantly inhibited cell proliferation and invasion in HNSCC cells [65]. Downregulation of miR-1 and upregulation of TAGLN2 were confirmed in HNSCC clinical specimens [65]. These data indicate that miR-1 is a tumor suppressive miR in HNSCC, and TAGLN2 may have an oncogenic function regulated by miR-1. This identification of novel miR-1-regulated cancer pathways could provide new insights into potential molecular mechanisms of HNSCC carcinogenesis. Another study found miR-1 expression is significantly downregulated in thyroid carcinomas as compared to normal thyroid tissue, suggesting miR-1 may also play a role in thyroid carcinogenesis [66].

### 4.5. miR-1 in Sarcoma

Sarcoma is a malignant tumor which forms in the connective or supportive tissues of the body, including bone, cartilage, fat, muscle, or blood vessels. Most miR-1 and sarcoma studies were on rhabdomyosarcoma (RMS). RMS is the most common soft tissue sarcoma in the pediatric malignancies and is classified into embryonal, alveolar, and pleomorphic RMS based on histological features [67]. Studies have found that many miRs are dysregulated in RMS [68]. Of those miRs, miR-1, miRNA-206, miRNA-133a, and miRNA-133b are the members of the muscle-specific miRs and often show decreased expression in RMS. Downregulation of miR-1 in RMS has been confirmed by northern blot analysis. Overexpression of miR-1 may inhibit rhabdomyosarcoma cell migration and proliferation through apoptosis and arrest cell cycle* in vitro* and restrain tumor growth* in vivo* [69]. More recent studies have shown that transfection of miR-1 in rhabdomyosarcoma cells suppressed c-Met expression, leading to inhibited cell proliferation and tumor growth [70, 71]. Elevated levels of PAX3 and cell proliferation genes are characteristic features of RMS [72]. Studies have found downregulation of miR-1 stabilizes the expression of PAX3 and CCND2 in both embryonal (ERMS) and alveolar (ARMS) RMS types. Transfection of miR-1 in ERMS cell line showed significant downregulation of PAX3 protein expression [69]. miR-1 is a potential regulator of PAX3 expression by binding to its 3′UTR. PAX3 plays a critical role in myogenesis, and increased expression of PAX3 is implicated in the pathogenesis of RMS. Specifically, in ARMS, fusion transcript PAX3-FOXO1 produces a protein product, within which the structural integrity of both PAX3 DNA-binding regions, the paired box and homeodomain, is retained. However, the 3′ UTR region of PAX3 is lost during the formation of this functional fusion transcript [72]. In this context, the loss of 3′UTR region of PAX3 due to formation of fusion transcript may allow the fusion transcript PAX3-FOXO1 to escape miR-1-mediated regulation. The author proposed that loss of 3′UTR of PAX3 and/or the downregulation of miR-1 are oncogenic events in rhabdomyosarcomagenesis. miR-1 can also regulate the expression of CCND2, a cell cycle gene [72]. Elevated CCND2 levels are observed in various cancers and are implicated in cell proliferation. miR-1 also regulates CCND2 transcript and protein levels. miR expression in chordoma-derived cell lines and chordoma tissue has been reported recently by using miR microarray technology [14]. Chordoma is an unusual rare tumor of bone in which the traditional treatment of surgical resection and radiation is often difficult, and there are no effective systemic therapies available [14]. Human chordoma tissues and cell lines can be distinguished from normal muscle tissue by comparing miR expression profiles. Expression of miR-1 was markedly decreased in both chordoma tissues and cell lines. When chordoma cell lines were transfected with miR-1, downregulation of known miR-1 targets was observed. These targets included Met and HDAC4 two genes that were observed to be overexpressed in chordoma. These results demonstrate that miR-1 may have a functional effect on chordoma tumor pathogenesis [14, 73]. miR-1 has also been found to be downregulated in osteosarcoma cell lines [74].

## 5. Therapeutic Potential of miR-1 Modulation

**Figure 3:**
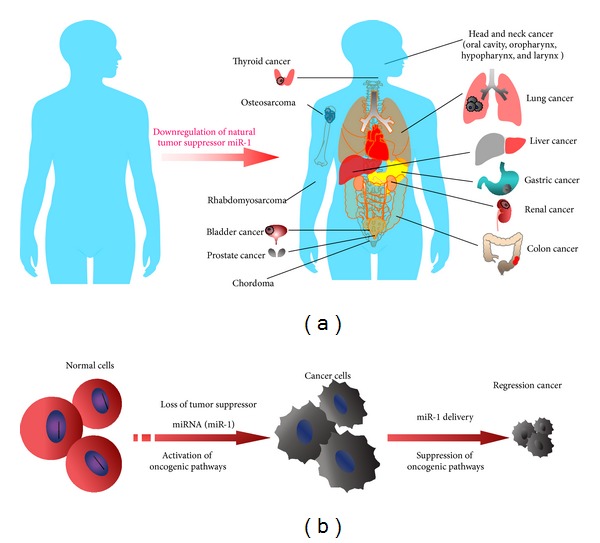
Aberrant expression of miR-1 in human cancers and its therapeutic potentials. (a) Association of decreased expression of miR-1 in different human cancers. (b) Loss of a tumor suppressor miR-1 leads to activation of oncogenic pathways and tumorigenesis. Delivery of miR-1 into the cytoplasm of tumor cells targeting oncogenes and subsequently suppressing tumor growth.

The rationale for exploring the therapeutic potential of miR-1 is based on the observation that a single miR-1 can regulate multiple oncogenes and oncogenic pathways that are commonly deregulated in different cancers (Figure 3). Several experimental approaches can identify actual miR-1 target genes in tumor cells. By using bioinformatic prediction programs, qRT-PCR, western blotting, and reporter assays, recent investigations have identified several oncogene and oncogenic pathway targets of miR-1 in different human cancers (Table 2). These targets potentially contribute to specific functional readouts of miR-1 as described above. For example, enforced expression of miR-1 in lung, colon, RMS, and chordoma cells dramatically suppressed Met expression and inhibited tumor cell growth [42, 44, 69]. Similar results were found in other types of cancer [14, 38].

miR-1 is consistently dysregulated across many cancers. The dysregulated miR-1 is not limited to a particular tumor type and, in some cases, the aberrantly expressed miRs correlate with clinical status such as the tumor stage and patient survival. Additionally, recent studies have observed a functional contribution of miR-1 to cellular transformation, tumorigenesis, apoptosis, and drug sensitivity. miR-1 provide critical functions downstream of classic oncogenic signaling pathways such as those controlled by Met, HDAC4, PIM-1, Wnt, Cyclin D, FOXP1, Slug, and TAGLN2. Finally, functional studies have directly documented the potent antitumorigenic activity of miR-1 both* in vitro* and* in vivo*. These exciting findings not only improve our understanding of the molecular mechanisms of human cancer but also provide a new class of potential molecular targets. As aberrantly expressed miR-1 plays key roles in the development of human cancer, correcting miR-1 dysregulation and deficiencies by restoring miR-1 function will provide a significant advance in cancer therapy.

Downmodulation of miR-1 has been identified as one of the events that enhance colorectal cancer progression [44]. miR-1 has tumor suppressor functions in colorectal cancer by directly downregulating MET oncogene both at RNA and protein level. Restoration of miR-1 expression leads to MET-driven reduction of cell proliferation and motility [44]. miR-1 has also been shown to act upstream from *β*-catenin of the canonical Wnt pathway [75]. The canonical Wnt pathway includes a pivotal cytoplasmic complex composed of APC, GSK3*β*, and Axin.**β**-Catenin may be phosphorylated by this complex and then degraded after ubiquitination. Activation of canonical Wnt signaling may cause accumulation of *β*-beta-catenin in the cytoplasm and then translocate to the nucleus [76]. Aberrant regulation of the Wnt signalling pathway has emerged as a prevalent theme in cancer biology, indicating that activation of this pathway may play an important role in tumor development. As Wnt/**β**-catenin signaling is tightly regulated at multiple cellular levels, this pathway offers many targeting nodal points for cancer drug development. miR-1 inhibits cell proliferation and viability during selection of human colon cancer cell lines that exhibit dysregulated Wnt signaling. By transducing miR-1 expressing lentiviruses into primary mammary organoids derived from muscle from which these tumors arise, reexpression of miR-1 rescued the myogenic differentiation program and blocked the tumorigenic phenotype [71]. These data support the notion that miR-1 holds substantial clinical potential as differentiation therapy for RMS and perhaps other solid tumors. miR-1 reexpression therapy constitutes a novel approach to inhibit oncogenes and arrest tumor development [77]. In lung cancer, reexpression of miR-1 induced apoptosis in cancer cells in response to the potent anticancer drug doxorubicin [75]. Enhanced activation of caspases 3 and 7, cleavage of their substrate PARP-1, and depletion of antiapoptotic Mcl-1 contributed to the sensitivity of miR-1-expressing cells to doxorubicin. Conductin-lacZ mice significantly reduced the expression of the Wnt-sensitive *β*-gal reporter [75]. These findings suggest the potential use of Wnt-modulating miR-1 as diagnostic and therapeutic tools in Wnt-dependent cancers, such as colorectal cancer. More recently, Slug has been identified as a miR-1 target in lung cancer A549 cells by TargetScan and picTar and a luciferase reporter assay with plasmids containing luciferase-Slug 3′-UTR [37]. These results suggest that reexpression of miR-1 may be an effective therapy that prevents cancer malignancy by converting cells from a mesenchymal phenotype to an epithelial phenotype via the downregulation of Slug. Moreover, induction of artificial transcription factor (ATF) expression in breast cancer* in vivo* by doxycycline resulted in 50% reduction in tumor growth and completely abolished tumor cell colonization [78]. Interestingly, ATF cDNA induction led to the reactivation of tumor suppressive miRs such as miR-1 [78].

In order for miR-1 to be effective as a cancer target, the therapeutic agents must be administered systemically, allow for specific and efficient delivery to the tumor tissue, and be taken up by tumor cells into the cytoplasm, where they can be available in intact form. While use of miR-1 has been effective for short-term gene inhibition in mammalian cell lines, its use for stable long-term transcriptional knockdown of target genes has been shown to be problematic [79, 80]. Similar to other nucleic acid constructs, miR-1 has stability and delivery problems for clinical applications. Like siRNAs, miRs have poor cellular membrane permeability and limited stability* in vivo* and intracellular delivery in tumor cells upon systemic administration [21, 22]. In order to translate miR-1 from an experimental approach to a clinical-viable therapeutic strategy that can benefit cancer patients, a specific and efficient delivery system is needed. Currently, plasmid and viral-based vectors are being used for miR delivery [21, 22]. Despite their high transduction efficiency, viral delivery approaches face serious challenges. Several gene therapy trials based on viral delivery systems have produced adverse effects questioning their safety [81, 82]. In addition, high costs for producing large amounts of viral stocks for clinical use and limited quantities of nucleic acids that can be packaged for therapy also limit the applications of viral delivery systems. Therefore, developing safe and effective nonviral miR-1 delivery systems is very important. Recently, a robust method for delivering unmodified miRs into cells has been developed by using cysteamine-functionalized gold nanoparticles (AuNPs) [83]. The efficiency of this method has been validated in two different tumor models and it showed that the best formulation of miR(1)-AuNP(10)-S-PEG(0.5) had the highest payload (10–20-fold higher than lipofectamine, a toxic transfection reagent for miRs* in vitro*), lowest toxicity (98% of cell viability following treatment), efficient uptake (96% uptake rate), fastest endosomal escape, and increased half-lives (at least 5 days) impacting cell proliferation and patterns of target gene expression [83]. These studies represent an essential step toward the advancement of nanotechnology-based miR delivery systems and evaluation of their therapeutic potentials in preclinical models.

## 6. Conclusion

There are now numerous examples linking dysregulated expression of miRs to cancer and its important role in human cancer. As reviewed above, miR-1 contributes to several biological processes of the tumor cell, including differentiation, proliferation, and apoptosis. Recently, studies from our group and others have shown that downregulation of miR-1 is one the most frequent events in some cancers. Herein, we have reviewed the expression of miR-1 and miR-1 regulated molecular targets in various types of cancer, and it is anticipated that further research will provide more convincing support for further miR-1 based therapy strategies.

## Figures and Tables

**Table 1:** Altered expression of miR-1 in different cancers.

Cancer type	miR-1 downregulation	miR-1 target genes	Reference number
Lung cancer	Primary lung cancer tissue and serum	MET; FoxP1; HDAC4; Slug; PIK3CA	[33, 36–38]
Colon cancer	Different colon cancer tissues	MET	[43]
Genitourinary cancer	Cancer cell lines and tissues	LASP1; TAGLN2; SRSF9; PTMA; PNP1	[50, 56, 57]
Head and neck cancer	Laryngeal carcinoma and MSSCC	TAGLN2; PTMA; FN1; PNP	[61, 62]
Thyroid cancer	Thyroid adenomas and carcinomas	CCND2; CXCR4; CXCL12	[66]
Hepatocellular cancer	HCC cell lines and tumor tissues	MET; FoxP1; HDAC4; ET-1	[39–41]
Sarcoma	Sarcoma cell lines and sarcoma tissues	MET; CCND2; HDAC4	[68, 69, 73]

**Table 2:** Oncogenes and oncogenic pathway targets of miR-1 in cancers.

Gene name	Genbank accession number	Chromosome location	Functions	Reference number
Met	NM_000245.2	7q31	Proto-oncogenic receptor tyrosine kinase	[42, 44, 68]
HDAC4	NM_006037.3	2q37.3	Histone deacetylase activity; represses transcription	[14, 31, 39]
Pim-1	NM_002648.3	6p21.2	Proto-oncogene	[33]
FOXP1	NM_001244810.1	3p14.1	Regulate gene transcription; tumor suppressor	[33, 39]
TAGLN2	NM_003564.1	1q21-q25	Earliest marker of differentiated smooth muscle	[50, 56, 64]
PNP	NM_000270.3	14q13.1	Purine metabolism	[50, 58]
PTMA	NM_002823.4	2q37.1	Enhance cell-mediated immunity	[58]
CXCR4	NM_001008540.1	2q21	Chemokine receptor	[65]
CCND2	NM_001759.3	12p13	Regulator of cyclin-dependent kinase	[65, 71]
SRSF9	NM_003769.2	12q24.31		[57]
FN1	NM_212482.1	2q34	Involved in cell adhesion, growth, and migration	[61]
ETS1	NM_001143820.1	11q23.3	Proto-oncogene	[40]
Endothelin 1	NM_001955.4	6p24.1	Involved in vascular disorders	[41]
Slug	NM_003068.4	8q11	Transcriptional repressor and has antiapoptotic activity	[38]
CXCL12	NM_199168.3	10q11.1	Chemotactic for lymphocytes	[65]

## Abbreviations

miR: MicroRNA
mRNA: Messenger RNA
miR-1: miRNA-1
UTR: Untranslated region.
